# Observations on the Use of Nitric Acid in Icterical Affections

**Published:** 1807-01

**Authors:** R. Hall

**Affiliations:** London


### *Observations on the Use of Nitric Acid in Icterical Affections.* By R. Hall, M. D. London.

SINCE the period when the nitric acid was first recommended as a remedy against syphilitic maladies, various experiments have been made, by different practitioners, with the view of ascertaining its curative influence in such affections. The results of these experiments seem to prove, that though this remedy be not competent to eradicate the disease in question, it is yet capable of restraining its progress, and improving the health and strength of the patient, in cases wherein, from the impaired state of the constitution, the introduction of mercury into the system would be obviously improper. While, therefore, it cannot be denied, that the nitric acid has not fully corresponded with the sanguine expectations of the individuals, by whom it was originally proposed as an antidote against lues venerea, it must, however, be admitted, that much benefit has resulted from the discussion thus excited, since by rousing the attention of the faculty to a remedy long and unaccountably neglected, they have been led to extend its use to a variety of other complaints, in which it has proved extremely beneficial.

Towards the conclusion of the last quarterly report of the diseases treated at the Carey-street Dispensary, I observe,* that the nitric acid has been administered in two cases of icterus; in both of which, Dr. Bateman remarks, the disease appeared to arise from the presence of gallstones, or inspissated bile, in the biliary ducts, as it was accompanied with frequent attacks of acute pain in that region. It was relieved, for a time, by opium, and calomel as a purgative; but in both, the most marked and essential benefit was derived from the use of the nitric acid. In one of the instances, the jaundice was speedily removed three or four successive times by this medicine, having returned when it was omitted for a short time; and in the other, not only the jaundice, but slight and frequent pains in the region of the liver, were invariably removed by recurring to its use. He farther mentions, that this remedy had been employed by him in a few other cases with similar advantage, as well as in some instances of chronic pains referred to the region of the liver.

---

* Edinburgh Medical and Surgical Journal, p. 498.

It affords me much satisfaction to be able to corroborate by my own experience, the above statement given by Dr. B. respecting the beneficial operation of the nitric acid in icterical affections. I myself was first induced to make a trial of it in such cases, in consequence of the analogy pointed out by Mr. Scott, of Bombay, in a communication to Sir Joseph Banks, between the action of this medicine and the supposed operation of mercury, which has proved so valuable a remedy in hepatic and icterical affections. No account of these cases was ever published; for my notes respecting them, as well as many others, were lost in my way to London during 1799; but I stated the result of my success in a note, inserted at page 61, of my translation of Guyton de Morveau, on the means of purifying infected air, &c. with a view to excite the attention of practitioners towards the subject.

The cases in which I had recourse to this mode of practice, were three in number, and occurred during the years 1797 and 1798. The patients were all females of sedentary habits, and considerably advanced in years. In two of the cases the disease was of no long standing; but in the third, the principal features of which I perfectly recollect, the complaint had existed for several months, and various remedies had been without effect employed to combat it. In this patient the skin and urine were extremely yellow, while what she voided by stool was whitish, or of a clay colour. She complained of frequent and acute pains at the scrobiculus cordis, sometimes darting back towards the spine, with a sense of weight in the right hypochondrium, accompanied with frequent vomitings. Her strength was much impaired, and her inferior extremities swelled towards the evening. Influenced by my success in the two former cases, as well as by the debilitated state of the patient, I determined to make a farther trial of the nitric acid in the present case. Of this medicine she was ordered to take to the extent of a drachm, in a state of proper dilution, daily. During the two or three first weeks of this course, there appeared very little progress towards amendment, but after increasing the dose of the acid to two drachms, her convalescence proceeded rapidly; the swelling of the inferior extremities no longer returned; her health and strength improved; her skin and other external parts of the body quickly lost their dingy yellow hue, and bile regularly appeared in the fæces. The pains also in the region of the stomach, and about the liver, which had formerly so much distressed her, as to render the use of opiates opiates occasionally necessary, now also abated; and, by a farther perseverance in the same remedy, her health, in about seven weeks from the period when she began to employ the nitric acid, was fully re-established. While I remained in the country, no return of the disorder occurred.

During the use of this medicine, there was an increased discharge of saliva, but no fœtor of breath; nor did the gums appear otherwise affected than by the local action of the acid. It seemed also to operate in promoting cutaneous perspiration, and in keeping the body open; for, previous to its use, the skin had been uniformly dry and parched, and the bowels in a torpid and constipated state. Since the above period, a single case has only fallen under my observation; but the patient became disgusted with the medicine, and notwithstanding I urged the propriety of its continuance, left off its use before any judgment could be formed as to its ultimate success. The complaint was afterwards removed by the sub-muriate of mercury.

What may be the modus operandi of the nitric acid in icterical affections, whether it acts as a solvent of biliary calculi, or operates by exciting such an action in the common biliary duct, or in that portion of the duodenum into which it enters, as to facilitate the discharge of such concretions, is difficult to determine. What seems, however, to countenance the former of these suppositions is, that, though from the violent and excruciating pain felt in the region of the liver, and from the obstructed excretion of bile, as well as other circumstances, there can, I think, be little doubt that biliary calculi existed in the above case; yet no such concretions were ever found among the fæces, though uniformly examined with the greatest care.

I have also, in common with other practitioners, witnessed very marked and essential benefit from the use of this acid in chronic hepatic obstructions, particularly in two cases, wherein the foundation of the complaint was laid in warm climates. As a tonic, in cases of general debility, and great mobility of the nervous system, as well as in typhous and other fevers, I have also derived the most unequivocal advantages from its exhibition.

Upon the whole, disposed however as I am to think highly of this remedy in various affections, it must yet be allowed, that farther and more numerous trials can alone furnish ground for forming a correct judgment of its real merit in the cure of icterus.

*Clement's Inn, Nov. 1, 1806.*

